# Barrett’s oesophagus and oesophageal cancer following oesophageal atresia repair: a systematic review

**DOI:** 10.1093/bjsopen/zrab069

**Published:** 2021-08-05

**Authors:** L Tullie, A Kelay, G S Bethell, C Major, N J Hall

**Affiliations:** Department of Paediatric Surgery and Urology, Southampton Children’s Hospital, Southampton, UK; National Institute for Health Research Great Ormond Street Hospital Biomedical Research Centre, University College London Great Ormond Street Institute for Child Health, London, UK; Stem Cell and Cancer Biology Laboratory, The Francis Crick Institute, London, UK; Department of Paediatric Surgery and Urology, Southampton Children’s Hospital, Southampton, UK; Department of Paediatric Surgery and Urology, Southampton Children’s Hospital, Southampton, UK; University Surgery Unit, Faculty of Medicine, University of Southampton, Southampton, UK; Department of Paediatric Surgery and Urology, Southampton Children’s Hospital, Southampton, UK; Department of Paediatric Surgery and Urology, Southampton Children’s Hospital, Southampton, UK; University Surgery Unit, Faculty of Medicine, University of Southampton, Southampton, UK

## Abstract

**Background:**

Concern exists that patients born with oesophageal atresia (OA) may be at high risk for Barrett’s oesophagus (BO), a known malignant precursor to the development of oesophageal adenocarcinoma. Screening endoscopy has a role in early BO identification but is not universal in this population. This study aimed to determine prevalence of BO after OA repair surgery, to quantify the magnitude of this association and inform the need for screening and surveillance.

**Methods:**

A systematic review, undertaken according to PRISMA guidelines, was preregistered on PROSPERO (CRD42017081001). PubMed and EMBASE were interrogated using a standardized search strategy on 31 July 2020. Included papers, published in English, reported either: one or more patients with either BO (gastric/intestinal metaplasia) or oesophageal cancer in patients born with OA; or long-term (greater than 2 years) follow-up after OA surgery with or without endoscopic screening or surveillance.

**Results:**

Some 134 studies were identified, including 19 case reports or series and 115 single- or multi-centre cohort studies. There were 13 cases of oesophageal cancer (9 squamous cell carcinoma, 4 adenocarcinoma) with a mean age at diagnosis of 40.5 (range 20–47) years. From 6282 patients under long-term follow-up, 317 patients with BO were reported. Overall prevalence of BO was 5.0 (95 per cent c.i. 4.5 to 5.6) per cent, with a mean age at detection of 13.8 years (range 8 months to 56 years). Prevalence of BO in series reporting endoscopic screening or surveillance was 12.8 (95 per cent c.i. 11.3 to 14.5) per cent.

**Conclusion:**

Despite a limited number of cancers, the prevalence of BO in patients born with OA is relatively high. While limited by the quality of available evidence, this review suggests endoscopic screening and surveillance may be warranted, but uncertainties remain over the design and effectiveness of any putative programme.

## Teaser

There are concerns that patients born with oesophageal atresia are at high risk for Barrett’s oesophagus, a known malignant precursor. This systematic review aimed to determine the prevalence of Barrett's oesophagus in patients born with oesophageal atresia under long-term follow-up to quantify the magnitude of the association and inform the need for screening. The prevalence of Barrett’s oesophagus is relatively high in this population, at 5 per cent, and suggests endoscopic screening may be warranted.

## Introduction

A number of reports have described oesophageal adenocarcinoma and squamous cell carcinoma (SCC) arising in adult survivors of surgery for oesophageal atresia (OA)^1–6^. The development of gastric and intestinal metaplasia in the oesophagus during childhood, adolescence or early adulthood has been widely documented^7–16^. These observations lead to the question of how these patients should be followed up to permit prompt detection of premalignant oesophageal mucosal changes. Currently, there is little consensus on either requirement for, or timing of, endoscopic screening or surveillance in patients born with OA.

Gastro-oesophageal reflux (GOR) is common following OA repair. The aetiology is probably contributed to by impaired oesophageal motility as well as disruption of the inherent antireflux mechanisms as a consequence of mobilization required to achieve an oesophageal anastomosis. The oesophageal mucosa may then be subjected to repeated exposure to refluxate that precipitates metaplasia. An international consensus statement has defined paediatric Barrett’s oesophagus (BO) as oesophageal metaplasia that is intestinal metaplasia positive or negative^17^.

Replacement of normal squamous epithelium in the distal oesophagus with columnar epithelium, as consequence of GOR, encompasses at least three different epithelial patterns. These are an intestinal type, usually harbouring mucous and goblet cells, as well as gastric fundus and cardiac types. Current evidence suggests that intestinal metaplasia represents the highest risk for subsequent dysplasia culminating in adenocarcinoma^18^. Controversy exists regarding the degree of malignant potential attributable to gastric metaplasia^19^.

BO is frequently occult and poorly correlated with the presence of reflux symptoms. One study reported no association between presence of symptoms of GOR in patients aged 15–19 years with and without histological evidence of BO^7^. Symptoms alone cannot be used to identify BO.

Whilst BO is well recognized following OA repair, the scale of the problem and associated morbidity has not been quantified beyond a handful of studies^13^^,^^20–22^. Without this evidence it is difficult to determine whether endoscopic screening and surveillance are indicated.

The primary aim of this review was to determine the prevalence of BO and oesophageal cancer in children, adolescents and adults born with OA to determine whether endoscopic screening and surveillance might be indicated. The secondary aim was to assimilate data to inform the design of any such surveillance programme in this population.

## Methods

This review was performed in accordance with the PRISMA guidelines for systematic reviews and according to a defined protocol registered with PROSPERO (York University, York, UK) prior to commencing the review (registration number: CRD42017081001)^23^^,^^24^.

### Search strategy

The search strategy was deliberately broad in order to be comprehensive and included studies reporting BO and/or oesophageal cancer in patients with repaired OA, in addition to those documenting long-term follow-up of patients born with OA. Several types of article were included to ensure that the search was systematic and that the findings would be as robust as possible. In addition to focusing on articles reporting outcomes of patients with OA, articles reporting cohorts of children having antireflux procedures or upper gastrointestinal endoscopy were also examined since these may have included patients born with OA. Searches were performed on 31 July 2020 using both the PubMed and Embase databases. In all databases, adjacency operators and truncation symbols were used in text word searches, when appropriate, to capture variations in phrasing and expression of terms. All synonymous terms were combined first using the Boolean ‘OR’. The three distinct concepts related to intervention, population and study design were combined with the Boolean ‘AND’. No language or date restrictions were applied. The detailed search strategy for each database used is included in *Fig. S1*, supplementary material. As well as using these databases, references in systematic reviews and randomized controlled trials, found in the search, were also included.

### Study inclusion criteria

Articles that met one or both of the following criteria were included: any study that reported at least one patient with BO or oesophageal cancer who had undergone either OA repair or oesophageal replacement having been born with OA; or any study that reported long-term follow-up (defined as minimum 2 years) of patients following OA repair or oesophageal replacement regardless of whether they included BO or oesophageal cancer, and regardless of the use of endoscopic screening (a single endoscopy) or surveillance (a programme of sequential endoscopies).

All study types were eligible for inclusion, including cohort studies and systematic reviews, with or without meta-analysis, and case reports. For the purposes of the search, a wide definition of BO was used that included any definition used by source article authors, including both gastric and intestinal metaplasia and heterotopic gastric mucosa.

### Study exclusion criteria

Studies were excluded if the patients had only an H-type tracheo-oesophageal fistula without OA. Studies were also excluded if they were abstracts only from conference presentations or published in non-English language. Where multiple reports from the same centre or authors were identified that resulted in duplication of cases or patient cohorts, either the first reporting study or the largest, in terms of patient numbers, was included.

### Article selection

Two reviewers independently assessed each title and abstract of all identified citations. Full-text articles were obtained if either reviewer considered the citation potentially relevant with a low threshold for retrieval. Full texts of selected studies were then reviewed critically to assess eligibility. Reasons for exclusion of studies were recorded. The final set of studies included in the systematic review was determined by consensus. The online resource Rayyan was used to assist with article screening and selection^25^. *A priori* it was decided not to use any risk of bias assessment tool and, as it was anticipated that all studies would probably be observational in nature, no study would be excluded based on methodology alone.

### Data extraction

Data were extracted independently, reviewed to ensure accuracy and entered into an electronic database recording paper title and author, study type, number of patients, length of follow-up, detail of endoscopic screening and/or surveillance and number of patients with BO/oesophageal cancer.

### Outcomes

The following outcomes were selected *a priori*: the number of patients with oesophageal cancer born with OA; the overall prevalence of BO and oesophageal cancer in patients born with OA; and the prevalence of BO and oesophageal cancer in patients born with OA who had undergone endoscopic screening or surveillance.

Further relevant clinical details of any patient with oesophageal cancer born with OA (such as age at diagnosis, type and site of cancer, detection method and outcome) were recorded if available, as were details of endoscopic screening or surveillance programmes and clinical details of patients with BO identified at endoscopy. For the purposes of reporting in this review, intestinal metaplasia was defined as metaplastic change alongside the presence of goblet cells and gastric metaplasia defined as metaplastic change without goblet cells.

### Statistical analysis

Data were entered and stored in an Excel (Microsoft, Redmond, Washington, USA) spreadsheet, descriptive analysis of data was undertaken using SPSS version 25 (IBM, Armonk, New York, USA). Data are reported as mean, median and range. The overall prevalence of BO and oesophageal cancer in patients born with OA was calculated by dividing the number of individuals with either BO or oesophageal cancer reported among the total population of OA patients by the total number of patients. The prevalence amongst the population who had undergone endoscopic screening or surveillance was calculated in a similar way, but limiting denominator population to those who had undergone one or more endoscopies.

## Results

### Characteristics of included studies

**Fig. 1:**
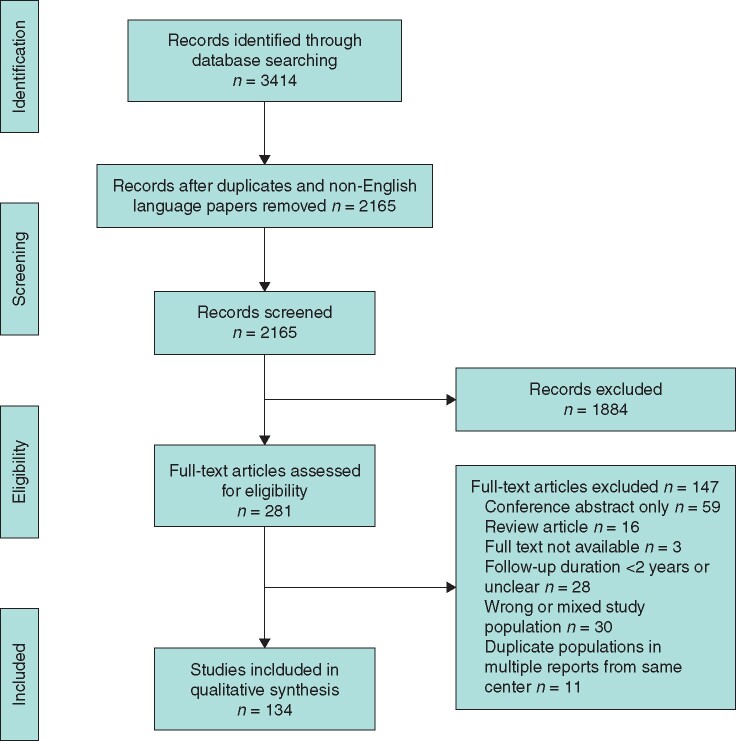
PRISMA diagram

A total of 134 articles met the inclusion criteria. Details of excluded articles are shown in *Fig. 1* including unavailability (3), conference abstract only (59), review article (16) and those which did not meet the inclusion criteria (58) involving short or unclear follow-up duration, wrong or mixed study population or disease process (such as oesophageal replacement in which OA and non-OA populations could not be separated). There were no cases of BO nor oesophageal carcinoma in these excluded studies. Populations published in multiple reports from the same centre were also excluded (11 populations)^26–36^.

The 134 articles were published between 1972 and 2020 and included 10 case reports and nine case series, reporting one or more cases of BO or oesophageal cancer in OA patients, and 115 either single- or multi-centre cohort studies, documenting long-term follow-up of OA patients with or without endoscopic screening or surveillance. These involved a total of 6282 OA patients with long-term follow-up (greater than 2 years) following either primary repair and/or oesophageal replacement. This total population figure was used as the denominator for the subsequent calculation of BO and oesophageal cancer prevalence. Median individual study population size was 87 (range 42–870) patients. The 6282 OA patients comprised both those who were documented to have undergone endoscopy during follow-up, including 1727 who had endoscopic screening or surveillance, and those who had not.

### Oesophageal cancer

There were 13 patients with oesophageal cancer identified in seven cohort studies and case reports from four centres in three countries (*Table 1*). Median age at diagnosis of oesophageal cancer was 40.5 (range 20–47) years; four were adenocarcinomas and nine SCCs. Five tumours were detected in the mid/distal oesophagus, three were adjacent to the site of the oesophageal anastomosis and two were in interposed segments replacing oesophagus (skin and colon)^1^^,^^2^^,^^5^^,^^6^^,^^37^. Three patients, two with adenocarcinoma and one with SCC, also had endoscopic evidence of BO^1^^,^^2^^,^^5^. There was one patient, with BO and low-grade dysplasia, in whom SCC was detected at surveillance endoscopy^5^.

**Table 1:** Reported cases of oesophageal malignancy following oesophageal atresia repair or replacement

Author	Setting and study type	No of patients	Age at diagnosis (years)	Malignancy type, site and grade	Clinical details	Outcome
**LaQuaglia *et al.* 1987^37^**	Case report (USA)	1	45	Squamous cell carcinoma Proximal oesophagus/skin tube T4N0M0	F, Gross type C Antethoracic skin tube conduit Non-smoker, no ETOH	Resection and colonic interposition Local proximal recurrence: re-resection and local radiotherapy
**Adzick *et al.* 1989^3^**	Case report (USA)	1	20	Adenocarcinoma Distal oesophagus/GOJ, T2N0M0	F, Gross type C Non-smoker, no ETOH No evidence of Barrett’s/oesophagitis	Oesophagogastrectomy and colonic interposition Alive at 1 year – no recurrence
**Deurloo *et al.* 2001^6^**	Case report (Netherlands)	1	38	Squamous cell carcinoma Mid-oesophageal (2 cm distal to previous anastomosis) T3N1M0	M, Gross type C Anastomotic stricture resection 18 mo Occasional smoker, 4 units ETOH/day	Neo-adjuvant chemotherapy Subtotal oesophagectomy and gastric tube interposition Postoperative radiotherapy Alive at 2 years – no recurrence
**Alfaro *et al.* 2005^2^**	Case report (USA)	1	46	Adenocarcinoma (Barrett’s and high-grade dysplasia) Mid-oesophagus Moderately invasive	F, primary repair	Neoadjuvant chemoradiotherapy Oesophagectomy and gastric transposition Alive at 2 months
**Pultrum *et al.* 2005^1^**	Case report (Netherlands)	1	22	Adenocarcinoma (and Barrett’s) At site of anastomosis T3N1M1 – moderate to highly differentiated	F, Gross type C Nissen fundoplication for GORD Endoscopic surveillance – no Barrett’s	Palliative radiotherapy and intraluminal stenting Died
**Jayasekera *et al.* 2012^5^**	Case series (Australia)	4	44, 46, 46, 44	Squamous cell carcinoma At site of anastomosis, T3N0M0 Mid/distal oesophagus (and associated sub-carinal mass) TXN2M0 SCC *in situ*, mid/distal oesophagus Mediastinal mass eroding through ribs and sternum	F, Gross type C Primary repair Heavy smoker 4 years (15–19 yo), non-smoker 25 years, no ETOH F, Gross type C Primary repair Non-smoker and no ETOH M, Gross type C 2× anastomotic stricture resection Smoker (20 pack years), 10g ETOH/week Barrett’s and low-grade dysplasia (annual surveillance for 10 years) M, Gross type C Repair of recurrent fistula and resection of stricture	Oesophagectomy, no chemoradiotherapy Recurrent local and metastatic disease 4 years later – died Chemoradiotherapy – ongoing at time of publication Unsuccessful endoscopic resection, ongoing chemoradiotherapy
**Vergouwe *et al.* 2018^4^**	Case series (Netherlands)	4	36, 42, 45, 47	Squamous cell carcinoma Distal oesophagus (25–32 cm) pT1bN0M0 Proximal oesophagus, with invasion of surrounding structures (trachea) T4N2M0 3 cm distal to anastomosis, pT2N0M0 Adenocarcinoma in colonic interposition pT2N1M0, moderately differentiated	F, Gross type A Primary repair (Livaditis elongation) Non-smoker and no ETOH M, Gross type A Delayed primary repair VACTERL Smoker, moderate ETOH M, Gross type C Primary repair Heavy smoker (27 pack years) and ETOH M, Gross type C Gastrostomy and oesophagostomy Colonic interposition (7 mo) VACTERL Smoker, minimal ETOH	Subtotal oesophagectomy, gastrectomy, colon interposition – metastatic disease at 12 months Chemotherapy (tumour unresectable) – alive at 6 years, no recurrence Oesophagectomy and gastric tube reconstruction. Further tumour in native cervical oesophagus 15 years later – died Chemotherapy, resection and gastric tube pull-up. Alive at 1 year

M, male; F, female; GOJ, gastro-oesophageal junction; ETOH, alcohol consumption; VACTERL, vertebral defects, anorectal anomalies, cardiac defects, tracheo-oesophageal fistula/oesophageal atresia, renal abnormalities and limb abnormalities; mo, months old; yo, years old.

At last recorded follow-up, five patients were alive, having completed treatment, five patients were receiving ongoing treatment and three had died (*Table 1*).

The overall prevalence of oesophageal cancer in OA patients under long-term follow-up was 0.002 per cent (13 of 6282 patients) with a prevalence of 0.06 per cent (1 of 1727) in the cohort who had undergone either endoscopic screening or surveillance.

### Barrett’s oesophagus

Some 317 patients with BO were reported in 48 cohort studies and case reports from 30 centres in 18 countries^7–10^^,^^12–16^^,^^20–22^^,^^38–73^, representing all reported patients with BO under long-term follow-up for OA.

Of these, intestinal metaplasia was identified in 54 patients, gastric metaplasia in 227, low-grade dysplasia in one, heterotopic gastric mucosa in three patients and type of metaplasia unspecified in 38.

The overall prevalence of BO in OA patients under long-term follow-up was 5.0 (95 per cent c.i. 4.5 to 5.6) per cent (317 of 6282 patients) (*Fig. S2*, supplementary material). The mean age at detection of BO was 13.8 years, median 16 years (range 8 months to 56 years).

### Endoscopic screening and surveillance

There were 1727 patients who underwent one or more endoscopies with or without biopsies during OA follow-up. The 24 studies in which either endoscopic screening or surveillance were undertaken are summarized in *Table 2* ^7–10^^,^^12–15^^,^^20–22^^,^^49^^,^^51^^,^^56–60^^,^^68^^,^^73–77^. They report endoscopies performed in defined OA populations with known numbers.

**Table 2:** Studies reporting endoscopic screening following oesophageal atresia repair or replacement

Author	Setting and study type	Population and age (range)	Intervention	Outcomes
**Ure *et al.* 1995^74^**	Single centre, prospective cohort	Long gap OA with colonic interposition 1963–1971 (*n* = 9) Mean 24 (22–27) years	UGIE + biopsies (*n* = 3)	0 cases of metaplasia or malignancy (0%)
**Somppi *et al.* 1998^15^**	Single centre, prospective cohort	OA repair/replacement 1963–1993 (*n* = 51)	UGIE + biopsies (*n* = 41)	2 gastric metaplasia (4.9%) Mean 12.6 (3.5–30) years
**Khan *et al.* 1998^75^**	Single centre, retrospective cohort	Colonic interposition for oesophageal replacement 1974–1993 (*n* = 25 of which OA *n* = 23)	UGIE + biopsies (*n* = 13)	0 cases of metaplasia (0%) (5–15 years)
**Krug *et al.* 1999^14^**	Single centre, prospective cohort	OA repair 1971–1978 (*n* = 39) (18–26 years)	UGIE + biopsies (*n* = 34)	2 intestinal metaplasia (5.8%)
**Deurloo *et al.* 2003^12^**	Single centre, prospective cohort	OA repair 1947–1972 (*n* = 38) Median 34 (28–45) years	UGIE + biopsies (*n* = 21)	1 intestinal metaplasia (4.8%)
**Deurloo *et al.* 2005^13^**	Single centre, prospective cohort	OA repair 1973–1985 (*n* = 92) Median 17 (10–26) years	UGIE + biopsies (*n* = 40)	3 gastric metaplasia (7.5%)
**Holschneider *et al.* 2007^49^**	Single centre, retrospective cohort	Fundoplications 1993–2005 (*n* = 160 of which OA *n = *87) Median 4.3 years (1 mo to 10 years)	UGIE + biopsies (*n* = 40)	1 intestinal metaplasia (2.5%)
**Taylor *et al.* 2007^51^**	Single centre, prospective cohort	OA repair before 1982 reviewed in clinic 2000–2003 (*n* = 132) Mean 33 (22–48) years	UGIE + biopsies (*n* = 62)	7 intestinal metaplasia (11.3%) of which 3 had concurrent low-grade dysplasia 1 squamous cell carcinoma^*^
**Castilloux *et al.* 2010^20^**	Single centre, prospective cohort	OA repair and >2 years old (or <2 years old and indication for UGIE) 2005–2008 (*n* = 45) Median 7.3 years (5 mo to 17 years)	UGIE + biopsies	16 gastric metaplasia (35.6%) Median 9.8 (3.4–13.2) years
**Sistonen *et al.* 2010^10^**	Single centre, prospective cohort	OA repair 1947–1985 (*n *= 98) Mean 36 (21–57) years	UGIE + biopsies	15 gastric metaplasia, 6 intestinal metaplasia (20.7%)
**Burjonrappa *et al.* 2011^9^**	Single centre, retrospective cohort	OA repair 1990–2009 (*n* = 51) Mean 6.6 years (7 mo to 19 years)	UGIE + biopsies (*n* = 38)	11 gastric metaplasia, 1 intestinal metaplasia (31.6%) Mean 13 years
**Pedersen *et al.* 2013^8^**	Single centre, prospective cohort	OA repair 1993–2005 (*n* = 59) Median 10.2 (5–15) years	UGIE + biopsies (*n* = 56)	1 intestinal metaplasia (1.8%)
**Huynh-Trudeau *et al.* 2015^56^**	Single centre, prospective cohort	OA repair/interposition with dysphagia (*n* = 41) Mean 25 (18–44) years	UGIE + biopsies (*n* = 32)	6 gastric metaplasia, 4 intestinal metaplasia (31.3%)
**Koziarkiewicz *et al.* 2015^57^**	Single centre, prospective cohort	OA repair 1990–2005 (*n* = 30) Median 13.7 (7–17) years	UGIE + biopsies (*n* = 12)	2 intestinal metaplasia (16.7%)
**Reismann *et al.* 2015^76^**	Single centre, retrospective cohort	Long gap OA treated with gastric transposition 1999–2012 (*n* = 9) Mean 6.2 (1.4–10.2) yeears	UGIE +/- biopsies (*n* = 8)	0 cases of metaplasia or malignancy (0%)
**Cartabuke *et al.* 2016^58^**	Single centre, retrospective cohort	OA repair/replacement 2011–2014 (*n* = 43) Median 8 (i.q.r. 3–20) years	UGIE + biopsies (*n* = 31)	2 patients Barrett’s oesophagus (type not specified) (6.5%)
**Gatzinsky *et al.* 2016^59^**	Single centre, prospective cohort	OA repair 1968–1983 (*n* = 29) Median 31 (25–40) years	UGIE + biopsies (*n* = 24)	2 intestinal metaplasia (8.3%)
**Iwańczak *et al.* 2016^60^**	Single centre, retrospective cohort	Thoracoscopic OA +/-TOF repair (*n* = 22) Mean 47 (16–79) months	UGIE +/- biopsies (*n* = 11)	1 gastric metaplasia (9.1%)
**Koivusalo *et al.* 2016^21^**	Single centre, retrospective cohort	Treated OA 1980–2014 (*n* = 211)	UGIE + biopsies (*n* = 209)	31 gastric metaplasia, 4 intestinal metaplasia (16.7%) Median 22 (16–32) years
**Schneider *et al.* 2016^7^**	Multicentre, prospective cohort	Primary OA repair (*n* = 120) Mean 16.5 (15–19) years	UGIE + biopsies (*n* = 120)	50 gastric metaplasia, 1 intestinal metaplasia (42.5%)
**Hsieh *et al.* 2017^22^**	Multicentre, retrospective cohort	OA followed up in specialist clinic (*n* = 541)	UGIE + biopsies	7 intestinal metaplasia (1.3%) Median 10 (2–17.2) years
**Vergouwe *et al.* 2018^68^**	Single centre, prospective cohort	OA patients 1948–1999 (*n* = 151) Median 25.4 (16.8–68.6) years	UGIE + biopsies (*n* = 151)	26 gastric metaplasia, 10 intestinal metaplasia (23.8%)
**Youn *et al.* 2018^77^**	Single centre prospective cohort	Gastric tube interposition (*n* = 25 with OA) Median 12 (3–18) years	UGIE + biopsies (*n* = 20)	0 cases of metaplasia (0%) Median 15.4 (9–18) years
**Petit *et al.* 2019^73^**	Single centre prospective cohort	OA patients 2005–2014 (*n* = 77) Median 4.9 (3.6–8) years	UGIE + biopsies (*n* = 73)	9 gastric metaplasia (12.3%) Median 2 years (1–3)

* Previously reported^5^. OA, oesophageal atresia; TOF, tracheal oesophageal fistula; UGIE, upper gastrointestinal tract endoscopy; VACTERL, vertebral defects, anorectal anomalies, cardiac defects, tracheo-oesophageal fistula/oesophageal atresia, renal abnormalities and limb abnormalities.

Twenty studies reported results of endoscopic screening; a single endoscopy to assess for BO, which was undertaken at mean age of 20 years (median 16 years (range 16 months to 57 years))^7^^,^^8^^,^^10^^,^^12^^–^^15^^,^^20^^,^^49^^,^^51^^,^^56^^–^^60^^,^^68^^,^^74^^–^^77^. While many of these studies, reporting screening endoscopies, suggested a requirement for further surveillance when BO was identified, few subsequently outlined their proposed surveillance regimen^68^.

Two studies reported the results of a combination of screening and surveillance endoscopies, but did not report the age range at which these were undertaken^22^^,^^73^. Two studies reported endoscopic surveillance in paediatric populations^9^^,^^21^. The first reported results from 3-yearly surveillance endoscopies from the age of 3 years until transition to adult care^9^. Additional ‘off-schedule’ endoscopies were undertaken in children with severe reflux in whom surgical intervention was under consideration^9^. In the second study, surveillance endoscopies were undertaken at 1, 3, 5, 10, 15 and over 15 years until the age of 17^21^.

There were 221 patients with BO (intestinal metaplasia, 49; gastric metaplasia, 170; metaplasia type unspecified, 2). The prevalence of BO in the cohort who had undergone endoscopic screening or surveillance was 12.8 (95 per cent c.i. 11.3 to 14.5) per cent (221 of 1727 patients) (range per series 0–42.5 per cent)^7–10^^,^^12–15^^,^^20–22^^,^^49^^,^^51^^,^^56–60^^,^^68^^,^^73–77^. Intestinal metaplasia was detected at a mean age of 38.5 years (median 38.5 (range 2-56) years) and gastric metaplasia at a mean age of 9.5 years (median 16.5 (range 2–56) years).

In those detected before the age of 16 years, identified by paediatric endoscopies, of the 49 patients with intestinal metaplasia, 11 were 15 years or younger and 38 were older than 15 years. Among those with gastric metaplasia, 60 patients were 15 years or younger and 101 were older than 15 years.

From studies reporting endoscopic surveillance, in six patients gastric metaplasia preceded intestinal metaplasia on sequential endoscopies, with gastric metaplasia occurring 1–5 years prior^21^^,^^22^. While there were two reported cases of resolution of BO (1 gastric and 1 intestinal) either spontaneously or following anti-reflux treatment, the majority of cases of BO persisted^9^^,^^47^^,^^66^. Gastric and intestinal metaplasia were present concurrently at screening endoscopy in four patients^22^. Three patients had intestinal metaplasia associated with low-grade dysplastic changes at screening endoscopy^51^. A single oesophageal cancer (SCC) was reported in the population who had undergone endoscopic surveillance^5^^,^^51^.

## Discussion

This systematic review identified a notable global prevalence of BO in this population, highest in those who had undergone endoscopic screening. Oesophageal cancer following OA repair or replacement remained rare, however, with just 13 patients reported, the majority of whom had SCC not adenocarcinoma. Only a single cancer (an SCC) was picked up by endoscopic surveillance.

The present review should be considered in the context of increasing concern that patients born with OA are at increased risk for developing oesophageal cancer^5^^,^^48^^,^^68^^,^^78^. Although the absolute number of cases of oesophageal cancer identified was relatively low, the likelihood of under-reporting seems considerable. The majority of studies reported follow-up in the paediatric period, in patients aged 15 years or younger, whereas all cancer diagnoses have occurred in adulthood with a mean age at diagnosis of 40 years. As there are no population-based cohort studies of patients born with OA being followed into adult life, it is not possible to define with certainty the true prevalence of oesophageal cancer in this population. The closest estimate is a population-based study from Finland of 272 patients born with OA with median 35 years of follow-up. No patients with oesophageal cancer were identified^11^. With a background incidence of oesophageal cancer in Finland at the time of 4.3 per 100 000 they were only able to exclude a prevalence of oesophageal cancer in patients born with OA of greater than 500 times that of the background population. Of note, patients in the present analysis developed oesophageal cancer at a younger age (median 40.5 years) than the general population, where the median age at diagnosis is around 64 years^79^.

BO is a recognized precursor to oesophageal adenocarcinoma, implying that endoscopic screening and surveillance of at-risk individuals, such as those with OA, might identify premalignant change and permit early interventions^80^. Based on the present review, an overall prevalence of BO in patients born with OA appears to be about 5 per cent in a mixed screened and unscreened population, rising to around 13 per cent in the screening and surveillance cohort. This is notably higher than the background prevalence of BO in both adult and paediatric populations, reported at 1.3–1.6 per cent and 0.002 per cent respectively^81–83^.

Despite this high prevalence, no patient under endoscopic surveillance progressed to adenocarcinoma. However, the majority of studies included in the review report cases of BO identified from screening rather than surveillance endoscopies. Although prevalence rates from screening suggest that endoscopic surveillance may be justified, it is unclear to what extent it would be either clinically beneficial or cost-effective.

A range of screening and surveillance programmes was identified in the present review. The youngest patient identified with BO (gastric metaplasia) was aged 8 months^42^. Intestinal metaplasia has been reported in a patient as young as 2 years^22^. In the present study, one in five cases of intestinal metaplasia and one third of gastric metaplasia cases, detected by endoscopic screening or surveillance, were in children aged 15 years or less. This may be taken to suggest that screening should start during childhood and, indeed, some authors have advocated that screening should commence during the teenage years or early 20s^7^^,^^9^^,^^48^. The optimal frequency of surveillance in this population also remains unclear. ESPGHAN guidance recommends three surveillance endoscopies during childhood in asymptomatic patients with treated OA: after stopping anti-reflux therapy, before the age of 10 years and a further endoscopy on transition to adult care^84^. Current adult guidelines recommend surveillance endoscopies every 2–5 years, depending upon the length and type of BO, with more frequent surveillance advised when dysplastic changes are present^19^^,^^85^.

In line with guidelines, the present review included both gastric and intestinal metaplastic change in the definition of BO^19^. This may explain why the prevalence of BO was as high as 43 per cent in one study^7^. Intestinal metaplasia is generally considered to be the significant risk factor for malignancy, specifically adenocarcinoma^86^, although the relative risks associated with gastric metaplasia, columnar epithelium without goblet cells, remains a subject of controversy^18^^,^^87^. The lack of documented progression of BO to oesophageal cancer in patients born with OA in the present review means the importance of either gastric or intestinal epithelial metaplasia in this population cannot be evaluated.

A notable observation in the present review was the preponderance of SCC rather than adenocarcinoma. The absence of a recognizable precursor lesion for SCC suggests that endoscopic surveillance based on BO would be ineffective. Until there is a sufficient number of high-quality studies with follow-up over a long time period, no firm conclusions can be drawn.

Despite the present study being limited by the quality of existing available evidence, the broad approach to identifying patients at risk and wide study inclusion criteria have proved informative. Few studies documented prospective endoscopic screening and surveillance programmes and this limits the ability to make comparisons between different screening or surveillance programmes. In view of the numbers involved, international collaborative studies should be undertaken to identify the optimal screening and surveillance programmes in this population and assess their clinical benefit and cost effectiveness.

## Supplementary Material

zrab069_Supplementary_Data
