# Supplementary material for: Barrett’s oesophagus and oesophageal cancer following oesophageal atresia repair: a systematic review
Source: BJS Open. 2021 Aug 5;5(4):zrab069. doi: 10.1093/bjsopen/zrab069 (PMC8405903; doi:10.1093/bjsopen/zrab069)
Supplement: zrab069_Supplementary_Data [file zrab069_supplementary_data.zip › Supplementary information.docx]

**Supplementary Figure 1. Search strategies utilised**

**Pubmed**

(((Oesophageal atresia[Title/Abstract]) OR (Esophageal atresia[Title/Abstract]) OR (Tracheoesophageal fistula[Title/Abstract])) AND ((Barrett’s oesophagus[Title/Abstract]) OR (Barrett’s esophagus[Title/Abstract]) OR (Malignancy[Title/Abstract]) OR (Dysplasia[Title/Abstract]) OR (Metaplasia[Title/Abstract]) OR (Neoplasia[Title/Abstract]) OR (Cancer[Title/Abstract]) OR (adenocarcinoma[Title/Abstract]) OR (Longterm[Title/Abstract]) OR (Long-term[Title/Abstract]) OR (Long term[Title/Abstract]) OR (Surveillance[Title/Abstract]) OR (Adult[Title/Abstract]) OR (follow up[Title/Abstract]) OR (adult[Title/Abstract]) OR (Followup[Title/Abstract]) OR (Follow-up[Title/Abstract]) OR (Outcome[Title/Abstract]) OR (Outcomes[Title/Abstract])))

**Embase**

( ( ABS ( "Oesophageal atresia"  OR  "Esophageal atresia"  OR  "Tracheoesophageal fistula" )  AND  ABS ( "Barrett's oesophagus"  OR  "Barrett's esophagus"  OR  malignancy  OR  dysplasia  OR  metaplasia  OR  neoplasia  OR  cancer  OR  adenocarcinoma  OR  longterm  OR  long-term  OR  "Long term"  OR  surveillance  OR  adult  OR  "follow up"  OR  followup  OR  follow-up  OR  outcomes  OR  outcom ) ) )  OR  ( ( TITLE ( "Oesophageal atresia"  OR  "Esophageal atresia"  OR  "Tracheoesophageal fistula" )  AND  TITLE ( "Barrett's oesophagus"  OR  "Barrett's esophagus"  OR  malignancy  OR  dysplasia  OR  metaplasia  OR  neoplasia  OR  cancer  OR  adenocarcinoma  OR  longterm  OR  long-term  OR  "Long term"  OR  surveillance  OR  adult  OR  "follow up"  OR  followup  OR  follow-up  OR  outcomes  OR  outcom ) ) )  AND  ( LIMIT-TO ( LANGUAGE ,  "English" ) )

**Supplementary Figure 2. Studies reporting long term follow-up, of patients born with OA, without report of oesophageal cancer or Barrett’s oesophagus***

*excludes articles already referenced in the original article

Laks H, Wilkinson RH, Schuster SR. Long-term results following correction of esophageal atresia with tracheoesophageal fistula: a clinical and cinefluorographic study. J Pediatr Surg 1972;7(5):591-7

Orringer MB, Kirsh MM, Sloan H. Long-term esophageal function following repair of esophageal atresia. Ann Surg. 1977; 186(4):436-43

O’Neill JA, Holcomb GW, Neblett WW. Recent experience with esophageal atresia. Ann Surg 1982; 195(6):739-45

Santos AD, Thompson TR, Johnson DE, Foker JE. Correction of esophageal atresia with distal trachoesophageal fistula. J Thorac Cardiovasc Surg 1983; 85(2):229-36

Lindahl H. Long-term prognosis of successfully operated oesophageal atresia-with aspects on physical and psychological development. Z Kinderchir 1984;39(1):6-10

Pineschi A, Pini M, Torre G, Levi N. Gastric tube oesophagoplasty for oesophageal atresia: a follow-up study. Part II: Radiologic, endoscopic and histologic controls. Z Kinderchir 1985;40(1):16-20

Luzzatto C, Ronconi M, Turra S, Guglielmi M, Zanardo V. Paediatr Padol 1990;25(5):313-20

Puri P, Ninan GK, Blake NS, Fitzgerald RJ, Guiney EJ, O’Donell B. Delayed primary anastomosis for esophageal atresia: 18months’ to 11 years’ follow-up. J Pediatr Surg 1992;27(8): 1127-30

Rescoria FJ, West KW, Scherer LR, Grosfeld JL. The complex nature of type A (long-gap) esophageal atresia. Surgery 1994;116(4):658-64

Lindahl H, Rintala R. Long-term complications in cases of isolated esophageal atresia treated with esophageal anastomosis. J Pediatr Surg 1995;30(8):1222-3

Engum SA, Grosfeld JL, West KW Rescoria FJ, Scherer LR. Analysis of morbidity and mortality in 227 cases of esophageal atresia and/or tracheoesophageal fistula over two decades. Arch Surg 1995;130(5):502-8

Macksood DJ, Blane CE, Drongowski RA, Coran AG. Complications after gastric transposition in children. Can Assoc Radiol J 1997;48(4):259-64

Foker JE, Linden BC, Boyle EM, Marguardt C. Development of a true primary repair for the full spectrum of esophageal atresia. Ann Surg 1997;226(4):533-41

Kimura K, Nishjima E, Tsugawa C, Collins DL, Lazar EL, Stylianos S, Sandler A, Soper RT. Multistaged extrathoracic esophageal elongation procedure for long gap esophageal atresia: Experience with 12 patients. J Pediatr Surg 2001; 36(11):1725-7

Schier F, Korn S, Michel E. Experiences of a parent support group with the long-term consequences of oesophageal atresia. J Pediatr Surg 2001; 36(4):605-10

Tomaselli V, Volpi ML, Dell’Agnola CA, Bini M, Rossi A, Indriolo A. Long-term evaluation of esophageal function in patients treated at birth for esophageal atresia. Pediatr Surg Int 2003;19:40-3

Calisti A, Oriolo L, Nanni L, Molle P, Briganti V, D’Urzo C. Mortality and long-term morbidity in esophageal atresia: the reduced impact of low birth weight and maturity on surgical outcome. J Perinat Med 2004;32(2):171-5

Seguier-Lipszyc E, Bonnard A, Aizenfisz S, Enezian G, Maintenant J, Aigrain Y, de Lagausie P. The management of long-gap esophageal atresia. J Pediatr Surg 2005;40(10):1542-6

Cimador M, Carta M Di Pace MR, Natale G, Catiglione A, Sergio M, Corsello G, De Grazia E. Primary repair in esophageal atresia. The results of long term follow-up. Minerva Pediatr 2006;58(1):9-13

Mastroianni R, Quaglietta L, Simeone D, Miele E, Capobianco A, Tramontano A et al. Respiratory symptoms and esophageal function after operation for esopahgeal atresia. Ital J Pediatr. 2007;33:330-35

Foker JE, Kendall Krosch TC, Catton K, Munro F, Khan KM. Long-gap esophageal atresia treated by growth induction: the biological potential and early follow-up results. Semin Pediatr Surg 2009;18(1)23-9

Hunter CJ, Petrosyan M, Connelly ME, Ford HR, Nguygen NX. Repair of long-gap esophageal atresia: gastric conduits may improve outcome-a 20-year single center experience. Pediatr Surg Int 2009;25(12):1087-91

Holland AJ, Ron O, Pierro A, Drake D, Curry JI, Kiely EM, Spitz L. Surgical outcomes of esophageal atresia without fistula for 24 years at a single institution. J Pediat Surg 2009;44(10):1928-32

Bax KM. Jejunum for bridging long-gap esophageal atresia. Semin Pediatr Surg 2009;18(1):34-9

Lacher M, Froelich S, von Schweinitz D, Dietz HG. Early and long term outcome in children with esophageal atresia treated over the last 22 years. Klin Paediatr 2010;222(5):296-301

Castilloux J, Noble AJ, Faure C. Risk factors for short- and long-term morbidity in children with esophageal atresia. J Pediatr 2010;156(5):755-60

Burgos L, Barrena S, Andres AM, Martinez L, Hernandez F, Olivares P, Lassaletta L, Tovar JA. Colonic interposition for esophageal replacement in children remains a good choice: 33-year median follow-up of 65 patients. J Pediatr Surg 2010;45(2): 341-5

Zhang Z, Huang Y, Su P, Wang D, Wang L. Experience in treating congenital esophageal atresia in China. J Pediatr Surg 2010;45(10):2009-14

Levin DN, Diamond IR, Langer JC. Complete vs partial fundoplication in children with esophageal atresia. J Pediatr Surg 2011; 46(5):854-8

Tannuri U, Tannuri AC. Should patients with esophageal atresia be submitted to esophageal substitution before they start walking? Dis Esophagus 2011;24(1):25-9

Aldabbagh, MH, Abdurrahman KN. Outcome of children in esophageal atresia with distal tracheoesophageal fistula who survived after repair: a case series of 20 cases. Pak Paed J 2012;36(3):158-63

Legrand C, Michaud L, Salleron J, Neut D, Sfeir R, Thumerelle C et al. Long-term outcome of children with oesophageal atresia type III. Arch Dis Child 2012;97(9):808-11

Oddsberg J, Lu Y, Lagergren J. Aspects of esophageal atresia in a population-based setting: incidence, mortality and cancer risk. Pediatr Surg Int 2012;28(3):249-57

Hayashi T, Inuzuka R, Shiozawa Y, Shindo T, Shimizu N, Katori T. Treatment strategy and long-term prognosis for patients with esophageal atresia and congenital heart diseases. Pediatr Cardiol 2013;34(1):64-9

Koivusalo AI, Parkarinen MP, Rintala RJ. Modern outcomes of oesophageal atresia: single centre experience over the last twenty years. J Pediatr Surg 2013;48(2):297-303

Maheshwari R, Trivedi A, Walker K, Holland AJ. Retrospective cohort study of long gap oesophageal atresia. J Pediatr Child Health 2013;49(10)845-9

Dingemann C, Zoeller C, Ure B. Thoracoscopic repair of oesophageal atresia: results of a selective approach. Eur J Pediatr Surg 2013;23(1):14-8

Lee HQ, Hawley A, Doak J, Nightingale MG, Hutson JM. Long-gap oesophageal atresia: comparison of delayed primary anastomosis and oesophageal replacement with gastric tube. J Pediatr Surg 2014;49(12):1762-6

Bardain S, Hamilton TE, Smithers CJ, Manfredi M, Ngo P, Gallagher D et al. Foker process for the correction of long gap esophageal atresia: Primary treatment versus secondary treatment after prior esophageal surgery. J Pediatr Surg

2015 Jun;50(6):933-7

Fragoso AC, Ortiz R, Hernandez F, Olivares P, Martinez L, Tovar JA. Defective upper gastrointestinal function after repair of combined esophageal and duodenal atresia. J Pediatr Surg 2015;50(4):531-4

Gallo G, Zwaveling S, Van der Zee DC, Bax KN, de Langen ZJ, Hulscher JB. A two-centre comparative study of gastric pull-up and jejunal interposition for long gap esophageal atresia. J Pediatr Surg 2015; 50(4):535-9

Uygun I, Zeytun H, Otcu S. Immediate primary anastomosis for isolated oesophageal atresia: A single centre experience. Af J Pediatr Surg 2015;12(4):273-9

Bakal U Ersoz F, Eker I, Sarac M, Aydin M, Kazez A. Long-term prognosis of patients with esophageal atresia and/or tracheoesophageal fistula. Indian J Pediatr 2016;82(5):401-4

DeBoer EM, Prager JD, Ruiz AG, Jensen EL, Deterding RR, Friedlander JA et al. Multidisciplinary care of children with repaired esophageal atresia and tracheoesophageal fistula. Pediatr Pulmonol 2016;51(6):576-81

Raitio A, Cresner R, Smith R, Jones MO, Losty PD. Fluoroscopic balloon dilatation for anastomotic stricture in patients with esopahgeal atresia: a fifteen-year single centre UK experience. J Pediatr Surg 2016;51(9):1426-8

Donoso F, Kassa AM, Gustafson E, Meurling S, Lijla HE. Outcome and management in infants with esophageal atresia – A single centre observational study. J Pediatr Surg 2016;51(9):1421-5

Bobanga ID, Barksdale EM. Foker technique for the management of pure esophageal atresia: long-term outcomes at a single institution. Eur J Pediatr Surg 2016;26(2):215-8

Coppens CH, van den Engel-Hoek L, Scharbatke H, de Groot SAF, Draaisma JMT. Dysphagia in children with repaired oesophageal atresia. Eur J Pediatr 2016;175(9): 1209-17

Zani A, Cobellis G, Wolinska J, Chiu PP, Pierro A. Preservation of native esophagus in infants with pure esophageal atresia has good long-term outcomes despite significant postoperative morbidity. Pediatr Surg Int 2016;32(2):113-7

Acher CW, Ostlie DJ, Leys CM, Struckmeyer S, Parker M, Nichol PF. Long-term outcomes of patients with tracheoesophageal fistula/esophageal atresia: survey results from tracheoesophageal fistula/esophageal atresia online communities. Eur J Pediatr Surg 2016;26(6):476-80

Awad K, Jaffray B. Oesophageal replacement with stomach: A personal series and review of published experience. J Paediatr Child Health 2017;53:1159-1166

Gibreel W, Zendejas B, Antiel RM, Fasen G, Moir CR, Zarroug AE. Swallowing dysfunction and quality of life in adults with surgically corrected esophageal atresia/tracheoesophageal fistula as infants: forty years of follow-up. Ann Surg 2017;266(2):305-10

Smithers CJ, Hamilton TE, Manfredi MA, Rhein L, Ngo P, Gallagher D et al. Categorization and repair of recurrent and acquired tracheoesophageal fistulae occurring after oesophageal atresia repair. J Pediatr Surg 2017;52(3):424-30

Zeng Z, Liu F, Ma J, Fang Y, Zhang H. Outcomes of primary gastric transposition for long-gap esophageal atresia in neonates. Medicine (Baltimore) 2017;92(26):e7366

Okuyama H, Tazuke Y, Uenoa T, Yamanaka H, Takama Y, Saka R et al. Long-term morbidity in adolescents and young adults with surgically treated esophageal atresia. Surg Today 2017;47(7):872-76

Madadi-Sanjani O, Zimmer J, Gosemann JH, Ure BM, Lacher M, Boehm R. Topical Mitomycin C Application in Pediatric Patients with Recurrent Esophageal Strictures-Report on Unfavorable Results. Eur J Pediatr Surg. 2018;28(6):539-546

Zhu H ,Wang M, Zheng S, Dong K, Xiao X, Shen C. Diagnosis and management of post-operative complications in esophageal atresia patients in China: a retrospective analysis from a single institution Int J Clin Exp Med 2018;11(1):254-261

Vergouwe FWT, van Wijk MP, Spaander MCW, Bruno MJ, Wijnen RMH, Schnater JM, et al. Evaluation of Gastroesophageal Reflux in Children Born With Esophageal Atresia Using pH and Impedance Monitoring. J Pediatr Gastroenterol Nutr. 2019;69(5):515-522

Leibovitch L, Zohar I, Maayan-Mazger A, Mazkereth R, Strauss T, Bilik R. Infants Born with Esophageal Atresia with or without Tracheo-Esophageal Fistula: Short- and Long-Term Outcomes. Isr Med Assoc J. 2018;20(3):161-166.

Lieber J, Schmidt A, Kumpf M, Fideler F, Schäfer JF, Kirschner HJ, et al. Functional outcome after laparoscopic assisted gastric transposition including pyloric dilatation in long-gap esophageal atresia. J Pediatr Surg. 2020 Jun 13:S0022-3468(20)30416-4. doi: 10.1016/j.jpedsurg.2020.06.004. Epub ahead of print.

Acharya SK, Sugandhi N, Jadhav AK, Bagga D, Tekchandani N, Sreedharan A, Srivastav S, Chakraborty G, Goel P. Gastric pull-up by the retrosternal route for esophageal replacement: Feasibility in a limited-resource scenario. J Pediatr Surg. 2020 Apr 28:S0022-3468(20)30300-6. doi: 10.1016/j.jpedsurg.2020.04.017. Epub ahead of print.

Jönsson L, Dellenmark-Blom M, Enoksson O, Friberg LG, Gatzinsky V, Sandin A, et al. Long-Term Effectiveness of Antireflux Surgery in Esophageal Atresia Patients. Eur J Pediatr Surg. 2019 Dec;29(6):521-527

Rayyan M, Embrechts M, Van Veer H, Aerts R, Hoffman I, Proesmans M et al. Neonatal factors predictive for respiratory and gastro-intestinal morbidity after esophageal atresia repair. Pediatr Neonatol. 2019;60(3):261-269

Jensen AR, McDuffie LA, Groh EM, Rescorla FJ. Outcomes for Correction of Long-Gap Esophageal Atresia: A 22-Year Experience. J Surg Res. 2020l;251:47-52

Bradshaw CJ, Sloan K, Morandi A, Lakshminarayanan B, Cox SG, Millar AJW, et al. Outcomes of Esophageal Replacement: Gastric Pull-Up and Colonic Interposition Procedures. Eur J Pediatr Surg. 2018;28(1):22-29

Platt E, McNally J, Cusick E. Pedicled jejunal interposition for long gap esophageal atresia. J Pediatr Surg. 2019;54(8):1557-1562. doi: 10.1016/j.jpedsurg.2018.10.108. Epub 2019 Jan 16.

Masuya R, Kaji T, Mukai M, Nakame K, Kawano T, Machigashira S et al. Predictive factors affecting the prognosis and late complications of 73 consecutive cases of esophageal atresia at 2 centers. Pediatr Surg Int. 2018;34(10):1027-1033

Lu YH, Yen TA, Chen CY, Tsao PN, Lin WH, Hsu WM et al. Risk factors for digestive morbidities after esophageal atresia repair. Eur J Pediatr. 2020 Jul 9. doi: 10.1007/s00431-020-03733-1. Epub ahead of print.

Slater BJ, Borobia P, Lovvorn HN, Raees MA, Bass KD, Almond S et al. Use of Magnets as a Minimally Invasive Approach for Anastomosis in Esophageal Atresia: Long-Term Outcomes. J Laparoendosc Adv Surg Tech A. 2019;29(10):1202-1206

Chiarenza SF, Bleve C, Zolpi E, Costa L, Mazzotta MR, Novek S, et al. The Use of Endoclips in Thoracoscopic Correction of Esophageal Atresia: Advantages or Complications? J Laparoendosc Adv Surg Tech A. 2019;29(7):976-980

Ganske IM, Firriolo JM, Nuzzi LC, Ganor O, Hamilton TE, Smithers CJ et al. Double Supercharged Jejunal Interposition for Late Salvage of Long-gap Esophageal Atresia. Ann Plast Surg. 2018;81(5):553-559

Foster JD, Hall NJ, Keys SC, Burge DM. Esophageal replacement by gastric transposition: A single surgeon's experience from a tertiary pediatric surgical center. J Pediatr Surg. 2018;53(11):2331-2335

Pellegrino SA, King SK, McLeod E, Hawley A, Brooks JA, Hutson JM et al. Impact of Esophageal Atresia on the Success of Fundoplication for Gastroesophageal Reflux. J Pediatr. 2018;198:60-66

Koivusalo A, Suominen J, Salminen J, Pakarinen M. Indications, Surgical Complications, and Long-Term Outcomes in Pediatric Esophageal Reconstructions with Pedicled Jejunal Interposition Graft. Eur J Pediatr Surg. 2020;30(1):111-116

Molino, J.A., Gine, C., Guillén, G. *et al.* Laparoscopic-assisted gastric pull-up: initial experience and technical details. *J Ped Endosc Surg* 2020;2:55–60

Takayasu H, Masumoto K, Sasaki T, Chiba F, Ono K, Gotoh C et al. Long-term follow-up in surgical newborns: A single-institution experience. Asian J Surg. 2020 Mar 19:S1015-9584(20)30073-7. doi: 10.1016/j.asjsur.2020.03.005. Epub ahead of print

Pani, E., Ciardini, E., Severi, E. et al. Thoracoscopic assisted colon interposition for long-gap esophageal atresia: a novel technique. J Ped Endosc Surg 2020;2:163–166

Son J, Jang Y, Kim W, Lee S, Jeong JS, Lee SK et al. Thoracoscopic repair of esopeahgeal atresia with distal TOF: is it a safe procedure in infants weighing <2000g. [Surg Endosc](https://www.ncbi.nlm.nih.gov/pmc/articles/PMC7222104/).2020;22:1–5

Stenström P, Anderberg M, Börjesson A, Arnbjörnsson E. Dilations of anastomotic strictures over time after repair of esophageal atresia. Pediatr Surg Int. 2017;33(2):191-195
